# Cytosolic Access of Intracellular Bacterial Pathogens: The *Shigella* Paradigm

**DOI:** 10.3389/fcimb.2016.00035

**Published:** 2016-04-05

**Authors:** Nora Mellouk, Jost Enninga

**Affiliations:** Dynamics of Host-Pathogen Interactions Unit, Institut PasteurParis, France

**Keywords:** *Shigella*, vacuolar rupture, intracellular pathogens, membrane trafficking, Rab GTPases

## Abstract

*Shigella* is a Gram-negative bacterial pathogen, which causes bacillary dysentery in humans. A crucial step of *Shigella* infection is its invasion of epithelial cells. Using a type III secretion system, *Shigella* injects several bacterial effectors ultimately leading to bacterial internalization within a vacuole. Then, *Shigella* escapes rapidly from the vacuole, it replicates within the cytosol and spreads from cell-to-cell. The molecular mechanism of vacuolar rupture used by *Shigella* has been studied in some detail during the recent years and new paradigms are emerging about the underlying molecular events. For decades, bacterial effector proteins were portrayed as main actors inducing vacuolar rupture. This includes the effector/translocators IpaB and IpaC. More recently, this has been challenged and an implication of the host cell in the process of vacuolar rupture has been put forward. This includes the bacterial subversion of host trafficking regulators, such as the Rab GTPase Rab11. The involvement of the host in determining bacterial vacuolar integrity has also been found for other bacterial pathogens, particularly for *Salmonella*. Here, we will discuss our current view of host factor and pathogen effector implications during *Shigella* vacuolar rupture and the steps leading to it.

## Introduction

Upon type 3 secretion system (T3SS)-triggered internalization into epithelial cells, *Shigella* rapidly ruptures its vacuolar membrane to reach the host cytosol for proliferation and cell-to-cell spread. Despite the importance of vacuolar rupture for intracellular bacterial proliferation and propagation, the underlying molecular mechanism has only recently been studied in more detail, and we still lack a precise understanding of the overall processes leading to and determining it (Ray et al., 2009, 2010; Carayol and Tran Van Nhieu, 2013a). Here, we will give an overview on the formation of the *Shigella*-containing vacuole, we will discuss the involvment of bacterial and host factors in the destablization of the vacuolar membrane, and we will compare it with vacuolar rupture by other bacterial pathogens.

## The steps leading to the formation of the *Shigella*-containing vacuole

*Shigella* internalization relies on elaborate plasma membrane and actin rearrangements, which are spatiotemporally controlled by the interplay between bacterial and cellular factors. Several *Shigella* T3SS effectors, namely IpaB/IpaC, IpgB1/2, and IpaD directly or indirectly modulate GTPase activation of the Rho family, including Rac1, Cdc42, and RhoA as well as host kinases to promote actin polymerization at the bacterial entry site. This leads to plasma membrane reorganization required for efficient formation of the *Shigella*-containing vacuole (Mounier et al., 1999; Carayol and Tran Van Nhieu, 2013a). Concomitantly, *Shigella* also alters the cellular levels of phosphoinositides (PIs) within targeted cells through the T3SS effector IpgD, thereby subverting several host pathways. IpgD is a phosphatidylinositol-phosphatase that specifically depletes PI(4,5)P_2_ resulting in the formation of PI(5)P (Niebuhr et al., 2002). IpgD is not required for bacterial entry, however it impacts on the way how the bacterium is internalized: Unlike the *Shigella* wildtype (WT) invasion site characterized by massive filopodia-like extensions and membrane ruffles, *ipgD* only induce a small albeit dense actin cup at the vicinity of the bacteria. Strikingly, ectopic expression of IpgD in epithelial cells results in a decrease in membrane-cytoskeleton tethering force and eventually causes membrane blebbing (Allaoui et al., 1993; Niebuhr et al., 2000, 2002; Mellouk et al., 2014). Therefore, it is likely that the depletion of PI(4,5)P_2_ by IpgD weakens plasma membrane-cortical actin interactions thereby facilitating membrane extensions (Saarikangas et al., 2010). In addition, IpgD-dependent production of PI(5)P during the internalization process of *Shigella* in epithelial cells recruits and activates the epidermal growth factor receptor (EGFR), independently of its bona fide ligand. In turn, EGFR activation stimulates the PI3K/Akt pathway activation. Importantly, PI(5)P production mediates a sustained Akt activation by promoting accumulation of active EGFR in early endosomes (EEs), protecting it from lysosomal degradation (Pendaries et al., 2006; Ramel et al., 2011). During these processes, PI(5)P allows the recruitment of the adaptor protein TOM1, which delays EGFR degradation and bulk endocytosis (Boal et al., 2015). Together, these findings suggest a primordial role of IpgD in the remodeling of the plasma membrane and the underlying actin cortex, as well as a regulator of lipid signaling during the communication of the *Shigella*-containing vacuole with its surrounding.

## Direct destabilization of *Shigella*-containing vacuoles by bacterial effectors

The step of vacuolar rupture takes place rapidly after bacterial internalization within epithelial cells. Studies that allow its tracking in real time revealed that the *Shigella*-containing vacuole gets damaged in <10 min (Paz et al., 2010; Ray et al., 2010). Early work implicated the T3SS effectors/translocators IpaB and IpaC in vacuolar rupture due to their ability to insert into the host cell membrane for the delivery of bacterial effectors. Collectively, these studies demonstrated that IpaB and IpaC can disrupt lipid vesicles (liposomes) *in vitro* and are required for contact-mediated hemolysis by *Shigella* as well as bacterial phagosomal escape in macrophages (High et al., 1992; Ménard et al., 1993; De Geyter et al., 1997; Blocker et al., 1999; De Geyter et al., 2000). In addition, IpaB was shown to be required for *Shigella*-induced macrophage death via direct binding and activation of caspase-1 (High et al., 1992; Hilbi et al., 1998). However, because IpaB and IpaC act both, as translocators and effectors, discriminating between these two functions represents a major experimental difficulty, both in macrophages and in epithelial cells. Furthermore, since bacterial uptake into epithelial cells also depends on a functional T3SS, it has been challenging to assess the direct contribution of IpaB and IpaC in the subsequent step of vacuolar escape in this cell type. Two recent studies provided new insight on IpaB function(s) using meticulous purification protocols allowing the preservation of its active conformation. Senerovic et al. showed that purified IpaB could oligomerize and form large channels, which would promote potassium influx at acidic pH. They further showed that IpaB-induced ions fluxes could activate caspase-1 via the inflammasome, thereby promoting macrophage pyroptosis. Intriguingly, the reported caspase-1/inflammasome-mediated death of macrophages required endocytosis of purified IpaB in a dynamin-dependent manner, however IpaB remained at the surface of epithelial cells (showing a defect of internalization) and thus did not affect their viability (Senerovic et al., 2012). In addition, Dickenson et al. showed that besides forming large channels, purified IpaB could also assemble into tetramers, and form small pore-like structures that presumably serve as scaffold for translocon insertion into membrane (Dickenson et al., 2013). Together, these works highlight distinct functions of IpaB, either serving as a structural translocator component for bacterial effector delivery into host cells or as a potent effector to induce endolysosomal leakage and promote macrophages pyroptosis. Noteworthy, T3SS translocators with high homology to IpaB and IpaC are present in a number of vacuolar-bound Gram-negative bacterial pathogens such as *Salmonella* and *Yersinia*. Moreover, contact-mediated hemolysis also occurs with *Yersinia* and *Salmonella* and also requires their respective translocator YopB and SipB (Håkansson et al., 1996; Hume et al., 2003; Coburn et al., 2007). Collectively, this suggests additional or other mechanisms controlling vacuolar membrane integrity during *Shigella* infection. For instance, the T3SS effector IpaH7.8 may promote *Shigella* vacuolar escape in macrophages, although its function is not yet established (Fernandez-Prada et al., 2000). Once *Shigella* gains access to the cytosol, vacuolar membrane remnants are polyubiquitinated, they recruit autophagic markers and adaptors such as LC3 and p62 as well as inflammasome components, and they are targeted to autophagic degradation, thereby dampening inflammatory response and promoting host cell survival (Dupont et al., 2009). LC3 has also been found surrounding the double-membranous vacuoles that *Shigella* forms after cell-to-cell spread, and two bacterial factors, namely IcsB and VirA have been implicated in the process of bacterial escape interfering with the LC3 recruitment (Campbell-Valois et al., 2015). Along these lines, constituents of the autophagy machinery have been shown to repair damaged vacuoles during *Salmonella enterica* infection (Kreibich et al., 2015). This indicates a link between the autophagy machinery, membrane repair, and their subversion through bacterial effectors, nevertheless an understanding of the underlying mechanism has remained illusive and requires further studies.

## Identification of host factors involved in *Shigella* vacuolar rupture

Compared to intravacuolar pathogens, considerably less is known about the interplay between cytosolic pathogens with the endocytic/exocytic pathways prior to their vacuolar escape, very likely because they rapidly escape into the cytosol and therefore were mostly assumed to not interact selectively with the host vesicular trafficking (Cossart and Roy, 2010; Fredlund and Enninga, 2014). Nonetheless a better understanding of the early events of vacuolar progression preceding cytosolic escape could provide novel insights into the pre-requisite for vacuolar rupture and potentially unravel new host factors subverted by bacterial pathogens to promote intracellular survival and proliferation. Noteworthy, *Listeria* has been shown to delay Rab5-GDP exchange and expression of constitutively active Rab5 induced bacterial degradation whereas dominant-negative Rab5 promoted bacterial survival and proliferation (Prada-Delgado et al., 2005). Albeit the mechanism of Rab5 modulation by *Listeria* is not yet established, it suggests that *Listeria* has evolved a strategy to avoid vacuolar maturation along the endolysosomal pathway prior to its escape into the cytosol.

To explore the involvement of host trafficking pathways in *Shigella* vacuolar rupture, an imaging-based vacuolar reporter assay was implemented in conjunction with siRNA screening (Ray et al., 2010; Keller et al., 2013). High-content/medium-throughput screens using an siRNA library targeting membrane traffic identified multiple host factors likely involved in *Shigella* uptake and vacuolar membrane rupture (Mellouk et al., 2014). In agreement with a subversion of the host cytoskeleton machinery to trigger bacterial entry, several host factors were found that promote actin polymerization such as the nucleator ARP2/3 complex and the Rho-GTPase Cdc42 (Tran Van Nhieu et al., 1999; Ehsani et al., 2012; Carayol and Tran Van Nhieu, 2013b). More surprisingly, a subset of endosomal factors was identified, particularly involved in sorting and/or recycling pathways notably including the EE markers Rab5 and EEA1, the sorting nexins (SNX1 and 2), and the recycling endosome markers Rab4 and Rab11. Accordingly, these findings indicate a complex mechanism underlying *Shigella* vacuolar escape that implicates the hijacking of several host pathways by the pathogen to efficiently gain access to the host cytosol, pinpointing a key role of endocytic and recycling regulators.

## Host membrane trafficking regulators and vacuolar rupture

The intravacuolar lifestyle requires the subversion of distinct endosomal and/or secretory pathways to diverge from the degradative lysosomal pathway and to build a unique niche for bacterial survival and proliferation. Accordingly, intravacuolar pathogens are well-described to selectively interact with host vesicles notably by hijacking key regulators such as Rab GTPases, SNAREs and PIPs to allow nutrient acquisition and vacuole expansion (Brumell and Scidmore, 2007; Weber et al., 2009; Cossart and Roy, 2010). Strikingly, altering the interplay of pathogen-containing vacuoles with the host membrane trafficking machinery has been shown to have dramatic consequences on their intracellular fate (Kumar and Valdivia, 2009; Creasey and Isberg, 2014). For instance, expression of either constitutively active form of Rab5 or dominant-negative form of Rab7 results in *Salmonella*-containing vacuole (SCV) disruption, implying that the Rab5-to-Rab7 conversion that promotes subsequent fusion with late endosomes (LEs) is crucial to maintain the SCV integrity (Brumell et al., 2002). Accumulating evidences suggest that *Legionella* manipulates multiple host cellular pathways using a wide range of type 4 secretion system effectors to promote intracellular growth in a partially redundant manner (Isberg et al., 2009; Hilbi and Haas, 2012). Two studies particularly support this emerging theme. Hoffmann et al. undertook a proteomic analysis of purified *Legionella*-containing vacuoles (LCVs) from macrophages and identified 9 Rabs (Rab1, 2, 4, 8, 10, 11, 14, 21, and 32) specifically localized on WT but not *icmT* mutant LCVs. Strikingly, subsequent knockdown of Rabs by siRNA showed that a subset of endocytic Rabs such as Rab5, Rab14, and Rab21 restrict intracellular replication whereas several secretory Rabs including Rab8, Rab10, and Rab32 had the opposite effect by promoting intracellular growth. Surprisingly, although numerous effectors target Rab1 function, knocking down Rab1 did not impair bacterial growth (Hoffmann et al., 2014). In this regard, O'Connor et al. implemented an innovative genetic screening approach that combines bacterial mutants and siRNA targeting of host factors to uncover the importance of functional redundancy of *Legionella* virulence. In particular, they revealed that the *Legionella* double mutant *LidA/WipB* exhibited intracellular growth defects concomitant with an increase in LCV disruption, ultimately promoting both bacterial degradation and macrophages apoptosis (O'Connor et al., 2012).

**Figure 1:**
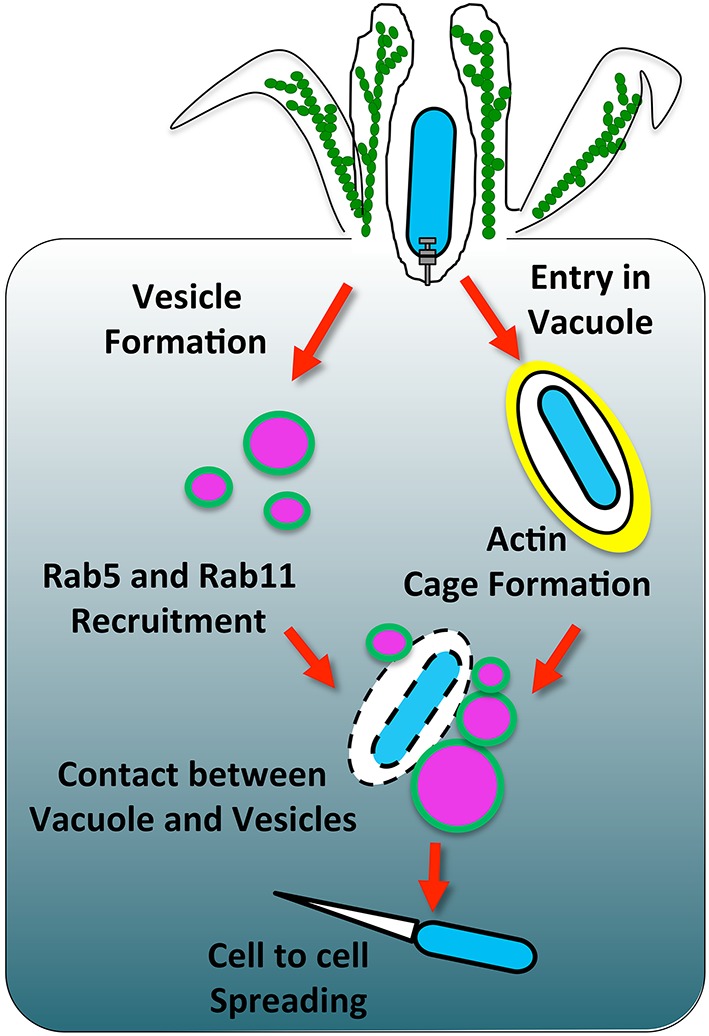
**The involvement of host membrane trafficking in *Shigella* vacuolar rupture**. *Shigella* induces its uptake into a vacuole that gets surrounded by an actin cage. Concomitantly, vesicles around the bacteria are modulated by IpgD to deplete PI(4,5)P_2_ and form PI(5)P (highlighted in green). This change of lipid content leads to the recruitment of the Rab GTPases Rab5 and Rab11. Vesicles around the *Shigella*-containing vacuole make contact with the bacterial compartment. During these contacts, the vacuole ruptures through a mechanism that requires further characterization.

As mentioned above, the siRNA screen on host factors involved in *Shigella* vacuolar escape suggested that endocytic and recycling pathways could be involved in the early stage of *Shigella* invasion (Mellouk et al., 2014). Live-imaging and quantification of the obtained data revealed the massive accumulation of Rab11-positive vesicles at the invasion site of *Shigella* prior to vacuolar rupture (Figure 1, left side). It also indicated the transitory presence of Rab5-positive vesicles whereas Rab4-positive vesicles were not enriched. Because *Shigella* rapidly escapes into the cytosol in less than 10 min, such accumulation of Rab11 at the invasion site was unforeseen (Paz et al., 2010; Ray et al., 2010). Indeed, although a number of intravacuolar pathogens including *Salmonella, Legionella*, and *Chlamydia* also recruit Rab11-positive vesicles, it commonly appears to be at an “intermediate or late” stage of vacuolar maturation presumably promoting bacterial proliferation by delivering nutrients and membrane to the growing bacterial vacuoles (Smith, 2005; Rejman Lipinski et al., 2009; Hoffmann et al., 2014).

Rab11 knockdown significantly delayed the vacuolar rupture of *Shigella* without disturbing its entry into epithelial cells (Mellouk et al., 2014). Interestingly, a genome-wide siRNA screen for *Chlamydia* infection in *Drosophila* cells where *Listeria* infection was also assessed in a secondary screen indicated that Rab11 knockdown inhibits both *Chlamydia* and *Listeria* infection (Derré et al., 2007). Later on, it was shown that both Rab6 and Rab11 knockdown prevent Golgi fragmentation induced by *Chlamydia*, thereby causing a defect in bacterial growth by impairing lipid transport to the inclusion (Rejman Lipinski et al., 2009). The preliminary results concerning *Listeria* require further investigations in a more physiological model, but it is tempting to speculate that the findings on Rab11 involvement in *Shigella* vacuolar rupture may be extended to other cytosolic pathogens.

## A link between Rab11 and PI subversion

Several pathogens hijack Rab11 cellular function, however we only start to understand the underlying mechanism for controlling Rab11 recruitment to the bacteria-containing vacuole (Guichard et al., 2014). Nevertheless, a widespread strategy used by intravacuolar pathogens to diverge from the degradative pathway and establish their replicative niche is the subversion of phosphoinositides (Weber et al., 2009). For instance, a key feature of *Legionella*-containing vacuole maturation is the accumulation of PI(4)P, most likely involving bacterial and host cellular phosphatases and kinases. In turn, PI(4)P anchors the effector DrrA (also called SidM), a Rab1-GEF required for Rab1 recruitment to the LCV (Machner and Isberg, 2006; Brombacher et al., 2009; Weber et al., 2013). *Mycobacterium* depletes PI(3)P by inhibiting the host PI3K hVPS34 and by the bacterial PI(3)P phosphatase, SapM thereby preventing further phagolysosomal maturation (Fratti et al., 2003; Vergne et al., 2005). In contrast, *Salmonella* generates PI(3)P on the SCV via the T3SS effector SopB to subsequently promote fusion with LEs. Although SopB may directly hydrolyse PI(3,4,5)P_3_ at the plasma membrane or PI(3,5) P_2_ on endosomes, it seems that SopB indirectly mediates PI(3)P accumulation on the SCV presumably by depleting PI(4,5)P_2_ to promote the recruitment of Rab5 and its effector hVPS34 (Marcus et al., 2001; Terebiznik et al., 2002; Hernandez et al., 2004; Mallo et al., 2008). Recently, Bakowski et al. showed that SopB depletion of PI(4,5)P_2_ increases the surface charge of the SCV, thereby preventing the accumulation of Rab8B, 13, 23, and 35 commonly targeted to the plasma membrane through their polycationic prenyl motif, and this potentially plays a role in the avoidance of the SCV-lysosome fusion (Bakowski et al., 2010). Collectively, bacterial pathogens thoroughly subvert PIs, which alter membrane identity subsequently affecting trafficking. Likewise, *Shigella* also modulates PIs through its effector IpgD, which acts as a phosphatidylinositol-phosphatase that specifically dephosphorylates PI(4,5)P_2_ into PI(5)P (Niebuhr et al., 2002). Results by us showed that the IpgD inositol phosphatase activity is absolutely required for the recruitment of Rab11-positive vesicles to the invasion site of *Shigella*. Using large volume correlative light and electron microscopy (CLEM), the ultrastructural details of the *Shigella* WT and *ipgD* invasion site were further characterized within epithelial cells and revealed that indeed Rab11-positive vesicles accumulate at the vicinity of the *Shigella* WT-containing vacuole but not around the *ipgD*-containing vacuole. More broadly barely any vesicles were observed at the *ipgD* invasion site, indicating that IpgD is not only required for Rab11 recruitment but also for the abundant presence of miscellaneous vesicles at the bacterial invasion site. The origin of these vesicles requires further investigations but based on their heterogeneous morphology we suggest that they could undergo successive fusion and fission events.

At the *Shigella* invasion site (characterized by membrane and actin rearrangements) it was found that: (i) PI(4,5)P_2_ was dramatically depleted in an IpgD-dependent manner, (ii) its precursor PI(4)P was enriched in an IpgD-independent manner presumably together with membrane ruffling rather than true enrichment, (iii) PI(5)P was enriched in an IpgD-dependent manner, and (iv) PI(3)P was not enriched in both cases (Mellouk et al., 2014). Secondly, at the *Shigella*-containing vacuole neither PI(4,5)P_2_, PI(5)P, PI(4)P, nor PI(3)P were found enriched, independently of IpgD. However, PI(3)P dynamically localized to a number of large vesicles present at the invasion site of *Shigella* WT but not *ipgD*, resembling PI(3)P-positive macropinosomes. These recent results corroborate the study from Niebuhr et al. where biochemical analysis revealed a global drop of PI(4,5)P_2_ cellular levels with a concomitant increase in PI(5)P controlled by the IpgD effector (Niebuhr et al., 2002). Furthermore, in our own studies we observed a certain enrichment of PI(5)P in the surrounding of the *Shigella*-containing vacuole but not particularly on its surface similarly to the previously reported data (Pendaries et al., 2006).

Yet in the case of *Salmonella*, SopB (homolog of IpgD) not only depletes PI(4,5)P_2_ at the plasma membrane but is also required for PI(4,5)P_2_ clearance at the surface of the SCV, thereby pinpointing a divergence between *Salmonella* and *Shigella* remodeling of PIs (Terebiznik et al., 2002; Mallo et al., 2008). In addition, despite the presence of large PI(3)P-positive vesicles at the invasion site, the *Shigella*-containing vacuole remains PI(3)P-negative. This corroborates the findings on Rab5 which is also transiently recruited at the invasion site without decorating the *Shigella*-containing vacuole (Mellouk et al., 2014). By contrast, as mentioned above *Salmonella* promotes the recruitment of Rab5 and the accumulation of PI(3)P on the SCV via SopB. Collectively, this suggested that *Shigella* diverges very rapidly from the classical endosomal (and intravacuolar pathogen) maturation by altering the PIP signature on the *Shigella*-containing vacuole and its surrounding trafficking notably by recruiting Rab11 and avoiding Rab5, ultimately leading to its vacuolar escape. Further studies are required to investigate the molecular mechanism of Rab11 recruitment via IpgD-mediated modulation and to decipher how *Shigella* avoids PI(3)P/Rab5, thereby shedding light on the intricate interplay between Rabs and PIPs.

## Actin surrounding the *Shigella*-containing vacuole and its rupture

In agreement with the fact that Rab11 promotes efficient vacuolar rupture and that *ipgD* completely abolishes the Rab11 recruitment to the invasion site, an *ipgD* mutant delays vacuolar rupture significantly. The *ipgD* mutant-containing vacuoles are predominantly surrounded by an F-actin meshwork we would like to coin an “actin cage,” which appears to confine the bacteria for extended periods of time. In contrast, *Shigella* WT-containing vacuoles are rarely observed confined within actin cages, suggesting that *Shigella* either prevents or rapidly disassembles the actin cage in an IpgD-dependent manner prior to vacuolar escape (Figure 1, right side). Previous observations of actin cages around *Shigella* WT-containing vacuoles have been reported, however the underlying mechanism controlling their dynamics remain uncovered (Ehsani et al., 2012). Given that PI(4,5)P_2_ dynamically controls numerous actin-binding proteins, the actin cage dynamics may be directly coordinated by IpgD modulation of the PI(4,5)P_2_ level at the invasion site. In particular, IpgD-mediated PI(4,5)P_2_ depletion is believed to be important for disrupting the linkage between the plasma membrane and cortical actin, thereby promoting membrane protrusions. In a similar manner, IpgD could prevent F-actin linkage to the vacuolar membrane, potentially by acting on cortical actin regulators such as the ERM-family proteins (Niebuhr et al., 2002; Fehon et al., 2010; Saarikangas et al., 2010). Remarkably, chemical depolymerization of the actin cage structure around the *ipgD* mutant-containing vacuole induces rapid rupture, further supporting that the actin cage confinement impairs efficient vacuolar escape (Mellouk et al., 2014). Noteworthy, *Salmonella* and *Chlamydia* promote the assembly of an F-actin meshwork resembling the actin cage structures observed in our study, which are crucial to maintain their vacuole integrity (Méresse et al., 2001; Kumar and Valdivia, 2008). Similarly, the actin cage could stabilize the *ipgD* mutant-containing vacuole by direct structural support.

## Vacuolar rupture by other bacterial pathogens

Interestingly, intravacuolar pathogens have evolved intricate strategies to maintain vacuolar integrity concomitantly with vacuolar expansion to accommodate bacterial replication. Similarly to the process of vacuolar rupture induced by cytosolic pathogens, the process of vacuolar integrity maintenance is not well-understood. However, it appears that the recruitment of potentially “destabilizing” host factors needs to be counterbalance to avoid vacuolar rupture (Kumar and Valdivia, 2009; Creasey and Isberg, 2014). For instance, numerous *Salmonella* effectors regulate the interaction of the SCV with cytoskeletal motors. In particular, PipB2 and SopD2 recruit kinesin-1 whereas SifA binds to the host SKIP to promote Sif formation. Importantly, in the absence of SifA, kinesin-1 accumulates on the SCV leading to vacuolar rupture (Beuzón et al., 2000; Boucrot et al., 2005; Dumont et al., 2010; Schroeder et al., 2010). In contrast, the *Salmonella* double mutant *sifA/sseJ* maintains vacuolar integrity, indicating that SseJ rather promotes vacuolar destabilization. SseJ is a phospholipase that promotes the depletion of cholesterol from the SCV, thus increasing membrane fluidity that may facilitate tubulation from the SCV but may also increase sensitivity to cytoskeleton motor-dependent forces leading to a loss of vacuolar integrity (Ohlson et al., 2005; Lossi et al., 2008). Likewise, *Legionella* secretes PlaA (homolog to SseJ), which also lead to vacuolar rupture in the absence of another bacterial effector, SdhA (Creasey and Isberg, 2012).

Vacuolar rupture induced by *Listeria monocytogenes* or *Rickettsia* was commonly believed to be directly and solely driven by bacterial effectors, notably through the pore-forming toxin listeriolysin O (LLO) and phospholipases (such as *Listeria* phospholipases C and *Rickettsia* phospholipases A and D) (Whitworth et al., 2005; Pizarro-Cerdá et al., 2012; Rahman et al., 2013). This has been changing due to an increasing number of reports revealing the implication of host factors in the process of vacuolar rupture for a number of bacterial pathogens. For instance, LLO-mediated *Listeria* vacuolar escape requires the host factors gamma-interferon-inducible lysosomal thiol reductase (GILT) and cystic fibrosis transmembrane conductance regulator (CFTR) to potentiate its activity (Singh et al., 2008; Radtke et al., 2011).

In contrast, the molecular mechanisms underlying *Rickettsia* and *Francisella* access to the host cytosol are poorly understood. *Rickettsia* species produce hemolysin C and phospholipases that seem to play a role in vacuolar escape (Renesto et al., 2003; Whitworth et al., 2005; Rahman et al., 2013). On the other hand, the *Francisella*-containing vacuole (FCV) subverts the endolysosomal route and harbors certain late endosomal markers but is devoid of lysosomal enzymes (Clemens et al., 2009). Acidification of the FCV is important for vacuolar escape and requires the host V-ATPase. Additionally, the host ubiquitin ligase CDC27 seems important to reach the cytosol, although the mechanism remains to be explored (Chong et al., 2008; Akimana et al., 2010). Furthermore, a subset of bacterial factors are involved in phagosomal escape, presumably by injecting some virulence factors such as IglI through a T6SS (Barker et al., 2009). Recently, Ramond et al. revealed that the *Francisella* glutamate transporter GadC plays a crucial role in phagosomal escape by neutralizing reactive oxygen species (ROS) production within the phagosome linking oxidative stress defense and phagosomal escape for this pathogen (Ramond et al., 2014).

Noteworthy, at late infection (12–18 h after its uptake into macrophages), *Legionella* is also released into the host cytosol prior to host cell lysis for bacterial dissemination (Molmeret et al., 2004). Recent studies have shown that *Mycobacterium* species can also escape from their phagosomal vacuole. In dendritic cells, a large portion of *M. tuberculosis* and *M. leprae* were found to reach the host cytosol in a T7SS-dependent manner after 2 days of infection (van der Wel et al., 2007). Furthermore, phagosomal escape of *M. tuberculosis* in macrophages was confirmed through the T7SS and especially requires the T7SS effector ESAT-6 (Simeone et al., 2012, 2015). Importantly, ESAT-6 has been directly implicated in vacuolar escape of the fish pathogen *M. marinum* by forming small pores into the vacuolar membrane (Smith et al., 2008).

## Conclusion

Collectively, it emerges that *Shigella* and other intracellular bacteria have evolved sophisticated ways to destabilize their vacuole to reach the host cytoplasm. The classic view of a process driven entirely by bacterial effectors or toxins has been amended by more recent findings putting the subversion of host processes in the spotlight. Here, the modulation of PIPs through dedicated effectors, such as SopB in the case of *Salmonella* and IpgD for *Shigella* that correlates with altered Rab GTPase recruitment to the bacteria-containing vacuoles drives the fate of this compartment. The precise molecular mechanisms that underlie these events remain to be studied. It is likely that deciphering this will provide fundamental information on how cells regulate endomembrane integrity.

## Author contributions

All authors listed, have made substantial, direct and intellectual contribution to the work, and approved it for publication.

### Conflict of interest statement

The authors declare that the research was conducted in the absence of any commercial or financial relationships that could be construed as a potential conflict of interest.
